# Harvard Odontological Society

**Published:** 1892-05

**Authors:** 

**Affiliations:** Harvard Odontological Society


### HARVARD ODONTOLOGICAL SOCIETY.

At the regular monthly meeting, held November 19, 1891, the President, Dr. Stanton, introduced the essayist of the evening, Dr. C. P. Briggs, of Boston, who read a paper entitled "Odontalgia."

(For Dr. Briggs's paper, see page 338.)

The discussion which followed was opened by Dr. E. C. Briggs, who said,—

As a contribution to the literature on this subject, the paper seems to be quite complete.

The question of diagnosis of toothache in the majority of cases is very simple, but we all have difficult presentations, and it is then that our knowledge is called upon, and sometimes it requires all we have to place the trouble where it belongs.

I have had examples of all the different cases that have been mentioned here to-night. In the closing up of the paper I noticed reference to the systemic diseases. I had a very interesting example of this variety recently. The patient was subject to rheumatic gout. He came with toothache, violent and agonizing. I felt helpless because I could do nothing to relieve; fortunately for me, the gout went to the back and left the tooth, and I escaped from making any mistakes.

I suppose all of us have had the experience of trouble with the second bicuspid resulting from the wisdom-tooth. I have met with it so often, that when a patient comes to me with pain in the superior second bicuspid, I immediately look at the lower wisdom-tooth on that side. Of course, I take a casual glance perhaps at the tooth itself, to see if it has any palpable sign of decay; but if it is not very evident that there is trouble there, I at once examine the lower wisdom-tooth.

In my experience with cases of diseases of the antrum, nothing pointed to the tooth as the cause. I remember being asked to examine a gentleman's mouth (by the throat specialist to whom he had gone for treatment) to see if there was any possible cause for disease in the antrum, and it was only by very careful examination that I located the trouble in an apparently sound tooth, which, on investigation, proved to be pulpless, and the treatment through that cured the disease.

The value of the paper, of course, is to draw our attention to the fact that we must keep well in mind the various causes, for the reason that we are apt, especially when the patient does not complain of very decided pain, and we find the teeth apparently right, to pass it off lightly. There is a tendency to do that, and the patient goes on suffering, when, if we would look into it more carefully, we might relieve the pain by finding the cause and effecting a cure. This amply repays one for the trouble taken.

*President Stanton.*—I am sure there must be a vast amount of experience here in regard to toothache; to me certainly it is a question of immense interest,—for this reason: a great many cases that we get in our office result in neuralgia, and to my mind neuralgia is a disease that, in the future, will be treated by dentists as much as by physicians. I think you find to-day that half the cases of neuralgia in and about the head and neck and face can be cured by a skilful dentist and according to his diagnosis. Such treatment also brings into play an anatomical knowledge of all parts of the head, of the eye, of the ear, and of the nose. Many times patients are sent to us by physicians who have failed to find the exciting cause in the face or scalp, and, after a careful examination of the teeth, we find they are correct as far as we can go; but by quizzing the patient we can ascertain something that the physician has overlooked, and at once relieve the trouble. That such cases are not uncommon, I feel confident; therefore, I think it is our duty, as well as a professional advantage, to become thoroughly familiar with this disease. To the knowledge which we should possess as good dentists we should add that of the different organs of the head, and if we bear in mind the mutual relations of the teeth and these organs, we shall understand many cases of neuralgia and treat them successfully.

In commenting upon a paper of this kind, one will necessarily have in mind some particular case of interest. I will describe that of a young lady who had been in perfect health all her life,—had never been troubled with rheumatism or neuralgia, but when she was about twenty-five years of age, she was taken ill with typhoid fever, and this left her with neuralgia, which, at various times, centred in her eyes, teeth, and right side of her head. It was a typical case, for which physicians had done all they could.

She had been to an optician for examination, and had been fitted with several pairs of glasses. She came to me and complained of pain in the left superior second bicuspid. On examination, I found there a small cavity, very superficial, which I filled with gold. The pain continued; I removed the gold, replacing it with cement; still the pain remained. I destroyed the pulp of the tooth and removed it, this giving relief for a number of months. All this time she had this general neuralgia of the scalp and eyes. Finally, after careful examination, I discovered that her glasses were not just right. I urged her to go to an oculist and have her eyes examined properly and carefully, and she was partially relieved; at the same time she insisted on the removal of the second bicuspid. I then made no objection, because I thought that if by the removal of the second bicuspid any of her pain could be relieved, that would be an excuse. I must confess, too, that I was curious to find out what there was in or about this bicuspid that was the probable cause of the trouble. I removed the tooth, and, on breaking it, found there a nodule of cementum that had developed to a considerable extent and was enlarging at the time. Now, my opinion is that in the sensitive condition of the nerves of her head, this small nodule was sufficient to centre the neuralgia in that tooth. The last I knew, she was comparatively comfortable, and I attributed her relief to the fact that the exciting cause had been removed by extracting the second bicuspid, and by having glasses properly adjusted to her eyes.

*Dr. Page.*—I was recently called out of town to the case of a woman who had been in bed six weeks, suffering with neuralgia. There were exposures in the second molars of the left side, superior and inferior. In these two teeth I made the usual applications, and the neuralgia, which her physician considered beyond the dentist's ken, vanished apparently by magic.

On Saturday last a case of tumor of the left side of the neck came to me. After an examination, I made an opening through the crown of the left inferior third molar, pus coming up through the opening. The tumor was removed by the contents being pressed through that opening in the crown of the third molar.

An improper occlusion is a cause of toothache, which I frequently find. In order either to confirm or eliminate this as a cause, let the patient bite into modelling compound or wax. Make a model from that and study it.

In many troublesome cases I find that no operation is needed save that of correcting the occlusion.

*Dr. Cooke.*—I remember an example in a right upper second molar which was very lame on percussion. The patient said it had ached all the previous day and night. There was no decay, and it did not look as if the pulp were dead. I could see no reason why it should have caused all that trouble. The wisdom-tooth behind was through the gum,—it did not articulate with the lower teeth, but was in close contact with the second molar. The wisdom-tooth was extracted; the patient had no more trouble, and the evidence was quite conclusive that the slanting of the third molar, acting as a lever upon the second molar, was the cause of the trouble.

*Dr. Grant.*—I will mention two cases that came to me about two years ago of fractured bicuspids (first superior). They had ached and were exceedingly sensitive; drawing an excavator between the two cusps was excruciating.

I put a simple gold band around them, and the teeth have been used continuously since; but the point of the excavator at the fractured line produces as great a shock as when the fractures first took place.

*Dr. Smith.*—Did you carry this gold band up under the gum, or was it simply a ferule?

*Dr. Grant.*—Just a narrow ferule, perhaps three-sixteenths of an inch wide.

*Dr. Smith.*—Doesn't the patient have any pain during mastication, or at any time, except when you place the excavator in the fracture?

*Dr. Grant.*—None whatever.

*Dr. Smith.*—The relation of cases is wise, I think, inasmuch as it adds to the sum-total of our experience, and gives us the opportunity of profiting by that of others. I had a case where the patient (male) had been treated by a physician, who had also called in another physician to assist him in his diagnosis. This patient had suffered for two weeks before I saw the case with severe neuralgia on the right side of his face, extending down the neck. He said he had "suffered the tortures of the damned," and I fancy he had, until finally the physician advised him to come to me in the hope that I might be able to relieve him. The mouth presented this appearance: The wisdom-tooth, the mesial portion of it, was struggling to get through; the second molar had an approximal mesial filling of cement, not very large; the sixth-year molar had been extracted. While the physicians hardly believed that the neuralgia came from his teeth, yet they thought possibly the erupting wisdom-tooth might be sufficient cause, and they had lanced that thoroughly and well. After talking with the patient some time, I concluded that the possible trouble was a pulp-stone in the second molar. I explained to him my suspicion, and advised him to have the cement filling removed and see what we could find. The filling was removed; I drilled carefully into the pulp-cavity and found there a little nodule of secondary dentine. It was detached, so it acted like a shot, resting right on the cornua of the pulp. I removed it and dressed the cavity. The patient came in the next Monday and thanked me very heartily for the relief he experienced. He said that Sunday night was the first night of rest he had had for two weeks, although receiving the combined skill of two physicians.

I had another case which was not exactly a case in practice, as it came to my notice through meeting on the street an acquaintance. She related to me her sufferings for six months, at frequent intervals, with terrible neuralgia of the shoulder, going down to the forearm. I asked her if her teeth had been examined, and she said that they had never been suspected by her physician as the cause of the trouble. I suggested that they might be, whereupon she called at my office. After a careful examination I found under the free margin of the gum, on the buccal side of the wisdom tooth, a cavity that penetrated to the pulp-chamber. It was such a difficult place to get at, and she had suffered so long, that I advised her to have it out, and after the extraction of that tooth the neuralgia disappeared from the shoulder.

I have seen other cases of neuralgia where apparently a very slight thing caused it all. I remember one case of a patient suffering with intense neuralgia, where there were no cavities in the teeth, except what had been well filled, but there was a noticeable recession of the gums, which I decided was the cause, from their hypersensitiveness. The exposed parts of the teeth were treated with a little nitrate of silver, which removed the sensitiveness and also the neuralgia.

I agree with you, Mr. President, that neuralgia has been of late, and will be in the future, more treated by dentists than formerly. One of our speakers has had all of his cases against the advice of physicians; many of mine have come to me by their advice. I speak of this as evidence that the physicians may be censured too severely. Elsewhere I think you will find, in most cases of neuralgia that are difficult of diagnosis, the patient is sent first to the dentist; and, in view of this, I think it is for our advantage, as you suggest, Mr. President, to be well informed on this subject, as by so doing we may not only reflect credit upon ourselves, but become of greater service to the human race.

*Dr. Page.*—I have spoken to you several times of a patient insane from toothache, the teeth apparently perfectly sound. Three times at intervals of about two years I have removed a pulp-stone. In each instance it has taken an hour or more to locate it. The attacks have been repeated four times altogether. In the first paroxysm he was so violent that it was necessary to strap him to the bed. The trouble then subsided without the cause being found. The second time the physician questioned me in regard to what I thought of it, and as a result, the patient was brought to my office in a carriage. After an hour's search for the cause, I made an application of arsenic, removed the pulp, treated the tooth, filled it, and he has the tooth to-day. About two years afterwards he was attacked in the same way and was again brought to my office, the trouble being on the opposite side, and was treated with like success. Two years afterwards he was again brought to me and again successfully treated. In every case the trouble was in the molar teeth, and in each instance relief was obtained inside of twenty-four hours. The patient is a man six feet one or two inches in height; not fat, but heavily built; his teeth are of the kind subject to pulp-stones,—very hard, short, and very yellow in color; the bucco-lingual diameter is greater than usual; the length of crown is shorter; the mesio-distal diameter is longer than usual, and they were considerably worn,—that is, the cusps were worn to the dentine. He grinds his teeth a great deal during sleep.

*Dr. Stoddard.*—I had a case that interested me greatly, from the fact that I was the patient myself. About the time of eruption of my wisdom-teeth, I had severe pain in the articulation of the inferior maxillary; sometimes the joint became so stiff that it would with difficulty close. The teeth were perfectly sound at the time, and I thought it due to neuralgia from eruption of the wisdom-teeth. Since that time I have seen a number of cases where perfectly sound wisdom-teeth have caused neuralgia at the period of eruption, although with not exactly those symptoms.

*Dr. Page.*—A year ago two very similar cases were sent to me by a physician. In the first one he said he feared lockjaw, and wished me to make an examination. The teeth were completely closed, and we did not dare to open them. In passing a probe along the side of the teeth, I came to the right inferior wisdom-tooth, and by the assistance of the physician, I pressed the cheek out and found what I thought was the edge of the gum over the wisdom-tooth. I took a platinum probe and passed it in there, a large amount of pus flowing out. I then passed in one end of a platinum tube (used in a syringe), tying the other end around a molar.

In a few days I removed pus enough to open the teeth, and found the third molar erupting in such a way that in biting, the right superior third molar pressed the gum over it, causing irritation and swelling, and this, together with the food fermenting there, resulted in suppuration. I removed part of the gum, used full strength aromatic sulphuric acid, gave a quantity to take home, with instructions how to use. The tissues healed, and the wisdom-tooth has since come through.

I have had cases similar to those described by Dr. Grant, and account for them in this way: a person bites something hard on the right side; the wedge-shaped cusps of the bicuspids on the left side strike each other; in the shock they are split apart, the noise at the time misleads, and one does not notice when it is done. It may also happen where one grinds his teeth while sleeping, one of the teeth occluding improperly.

I will cite one more case, that of a man, a wood-carver, who grated his teeth while sleeping to such a degree as to be *painfully* near neuralgia. I tried to evolve some scheme to mitigate the evil, finally devising a vulcanite covering to fit over both the upper and lower sets, armed with smooth surfaces.

He could put these on and grind away all night with no adverse results.

*Dr. Grant.*—How do you treat those cases of fractured teeth?

*Dr. Page.*—Make an arsenious acid application, remove the pulp, band, and fill the tooth.

HENRY L. UPHAM, D.M.D.,
*Editor Harvard Odontological Society.*

